# Inequalities in participation and time spent in moderate-to-vigorous physical activity: a pooled analysis of the cross-sectional health surveys for England 2008, 2012, and 2016

**DOI:** 10.1186/s12889-020-08479-x

**Published:** 2020-03-19

**Authors:** Shaun Scholes, Jennifer S. Mindell

**Affiliations:** Research Department of Epidemiology and Public Health, University College London, 1-19 Torrington Place, London, WC1E 7HB UK

**Keywords:** Physical activity, Moderate-to-vigorous intensity physical activity, Inequalities, Hurdle models

## Abstract

**Background:**

Evidence is unclear on whether inequalities in average levels of moderate-to-vigorous physical activity (MVPA) reflect differences in participation, differences in the amount of time spent active, or both. Using self-reported data from 24,882 adults (Health Survey for England 2008, 2012, 2016), we examined gender-specific inequalities in these separate aspects for total and domain-specific MVPA.

**Methods:**

Hurdle models accommodate continuous data with excess zeros and positive skewness. Such models were used to assess differences between income groups in three aspects: (1) the probability of doing any MVPA, (2) the average hours/week spent in MVPA, and (3) the average hours/week spent in MVPA conditional on participation (MVPA-active). Inequalities were summarised on the absolute scale using average marginal effects (AMEs) after confounder adjustment.

**Results:**

Inequalities were robust to adjustment in each aspect for total MVPA and for sports/exercise. Differences between adults in high-income versus low-income households in sports/exercise MVPA were 2.2 h/week among men (95% confidence interval (CI): 1.6, 2.8) and 1.7 h/week among women (95% CI: 1.3, 2.1); differences in sports/exercise MVPA-active were 1.3 h/week (95% CI: 0.4, 2.1) and 1.0 h/week (95% CI: 0.5, 1.6) for men and women, respectively. Heterogeneity in associations was evident for the other domains. For example, adults in high-income versus low-income households were more likely to do any walking (men: 13.0% (95% CI: 10.3, 15.8%); women: 10.2% (95% CI: 7.6, 12.8%)). Among all adults (including those who did no walking), the average hours/week spent walking showed no difference by income. Among those who did any walking, adults in high-income versus low-income households walked on average 1 h/week less (men: − 0.9 h/week (95% CI: − 1.7, − 0.2); women: − 1.0 h/week (95% CI: − 1.7, − 0.2)).

**Conclusions:**

Participation and the amount of time that adults spend in MVPA typically favours those in high-income households. Monitoring inequalities in MVPA requires assessing different aspects of the distribution within each domain. Reducing inequalities in sports/exercise requires policy actions and interventions to move adults in low-income households from inactivity to activity, and to enable those already active to do more. Measures to promote walking should focus efforts on reducing the sizeable income gap in the propensity to do any walking.

## Background

Being physically active increases cardiometabolic health and reduces risk of cardiovascular-related morbidity and mortality [1]. Inequalities in physical activity (PA) contribute to social gradients in health [2, 3]. However, previous studies on inequalities in adult PA have produced inconsistent findings due, at least in part, to heterogeneity in analysis techniques such as choice of PA indicator and whether assessment was made for total or domain-specific PA [4, 5].

The direction and/or magnitude of inequalities can also vary by whether PA is analysed as a binary, ordinal or continuous variable. Whilst enabling assessment with regard to PA recommendations, categorising a continuous variable such as the hours-per-week spent in moderate-to-vigorous PA (MVPA) into a binary [4, 6] or ordinal variable [7, 8] loses extensive information in the discretisation and is suboptimal both in terms of power and bias [9]. Yet analysing the continuous variable is also problematic. First, analyses based on the mean can mask inequalities in other parts of the distribution (e.g. at the lower- or upper-tails) [10]. Quantile regression facilitates assessment across continuous distributions; evidence suggests larger inequalities at the upper-tail of the body mass index (BMI) distribution [11, 12]. Secondly, MVPA distributions are not typically normally distributed but are characterised by excess zeros (persons not doing any) and positive skewness (high MVPA for a small number of highly active adults) [13], with each aspect potentially having different determinants [14].

Hurdle models such as those proposed by Cragg [15] can handle continuous MVPA data with excess zeros and positive skewness [13] as they treat participation and the amount of time spent active (conditional on participation) separately. Although used in the economics literature, especially for sports participation [14, 16], no epidemiological studies to date have used hurdle models to quantify inequalities in MVPA, despite the potential advantages for policy-makers and practitioners. Such advantages stem from simultaneously fitting separate model equations for participation (i.e. whether persons engage in MVPA or not) and for duration (i.e. the amount of time persons who do participate spend being active). By making this distinction, the results from hurdle models allow policy-makers and practitioners to evaluate whether factors such as income influence MVPA in the same or in opposite directions (e.g. higher income groups having higher participation rates but, conditional on participating, spending less time engaged) [14]. Such a distinction – suggesting policy actions and interventions be focused on increasing participation rather than increasing PA duration among those who are already active - is not possible using single equation models. Using nationally-representative health survey data, we applied hurdle models to quantify inequalities in total and domain-specific MVPA. We hypothesised that adults in high-income versus low-income households are more likely to participate in MVPA, and that conditional on doing any MVPA, spend more time on average being active.

## Methods

### Study sample

Data came from the Health Survey for England (HSE): this dataset is used to monitor progress on numerous national health objectives, including PA [17, 18]. Details about the HSE sample design and data collection are described elsewhere [19]. Briefly, the HSE annually draws a nationally-representative sample of people living in private households in England using multistage stratified probability sampling with postcode sectors as the primary sampling unit and the Postcode Address File as the household sampling frame. All adults in selected households are eligible for interview. Fieldwork takes place continuously through the year. Trained interviewers measured participants’ height and weight and assessed their demographic characteristics, self-reported health, and health behaviours including PA using computer-assisted personal interviewing. We used the most recent surveys (2008, 2012, 2016) that included the adult Physical Activity and Sedentary Behaviour Assessment Questionnaire (PASBAQ).

The household response rate ranged from 64% in 2008 to 59% in 2016. This study is restricted to adults (i.e. aged 16 years or over). Participants gave verbal consent for interview. Relevant committees granted research ethics approval for the survey. Overall, 31,399 adults participated in the three surveys, of whom 31,183 had valid PA data. Of these, 6301 had missing income data, leaving an analytical sample of 24,882 adults with complete data.

### Assessment of leisure-time physical activity

PASBAQ data is used to monitor adherence to UK PA recommendations [17, 18] and for other epidemiological research [20, 21]. The PASBAQ has demonstrated moderate-weak convergent validity in comparison with non-synchronous accelerometry [22]. PASBAQ assesses frequency (number of days in the last 4 weeks) and duration (of an average episode of at least 10 min) in four leisure-time domains [23]:

- “light” and “heavy” domestic activity;
- “light” and “heavy” manual work (e.g. ‘Do-It-Yourself’ (DIY));
- walking (with no distinction between walking for leisure or travel); and
- sports/exercise (ten specific and six ‘other’ activities).

“Heavy” domestic and manual activities were classed as moderately-intensive. Walking intensity was assessed by a question on usual walking-pace (responses: slow, average, fairly brisk, or fast); moderate-intensity was classed as a fairly brisk or fast pace. Intensity of sports/exercise was determined as indexed in the metabolic equivalent (METs) compendium [24, 25] and a follow-up question on whether the activity had made the participant “*out-of-breath or sweaty*”.

### Assessment of occupational physical activity

In addition to leisure-time PA, participants engaged in any paid or unpaid work answer questions on occupational PA. Our analyses classed three activities – walking, climbing stairs or ladders, and lifting, carrying, or moving heavy loads - as moderate-intensity PA for participants working in occupations identified a-priori as moderately-intensive [17].

### Assessment of time spent in MVPA

Time spent in domain-specific MVPA was calculated as the product of frequency and duration, converted from the last 4 weeks to hours/week. For sports/exercise, time in vigorous-intensity activities was multiplied by two when combined with moderate-intensity activities to calculate ‘equivalent’ hours/week as specified in MVPA guidelines [26]. Total MVPA was calculated by summing across the five domains (four leisure-time plus occupational), and was truncated at a maximum of 40 h/week to minimise unrealistic values.

### Socioeconomic position ascertainment and confounders

Household income was our chosen marker of socioeconomic position (SEP). The household reference person reports annual gross household income via a showcard (31 bands ranging from ‘less than £520’ to ‘£150,000+’). Household income is equivalised (McClements scale [27]), and grouped into tertiles. Age (in ten-year bands), current smoking (current, ex-regular, never), self-rated health (‘very good/good’, ‘fair’, or ‘bad/very bad’), and BMI were chosen as potential confounders of the SEP and MVPA associations [6]. We computed BMI as weight in kilogrammes (kg) divided by height in metres squared (m^2^), classifying participants into four groups according to the World Health Organization (WHO) BMI classification [28]: underweight (< 18.5 kg/m^2^), normal-weight (18.5–24.9 kg/m^2^), overweight (25.0–29.9 kg/m^2^), or obese (at least 30.0 kg/m^2^).

## Statistical analysis

### Descriptive estimates

Data was pooled over the three surveys to increase precision (prior analyses revealed no change in associations over time). Differences in age, self-rated health, current smoking, and BMI were estimated by income, using Rao-Scott tests for independence [29]. For total and domain-specific MVPA, we computed descriptive estimates for four outcomes:

- % doing any;
- % ‘sufficiently’ active (i.e. at least 2.5 h/week MVPA [26]);
- average hours/week MVPA (range: 0 to 40 h/week); and
- average hours/week MVPA among those doing any (range: 0.042 to 40 h/week; hereafter referred to as MVPA-active).

Outcomes MVPA and MVPA-active represent unconditional and conditional (on participation) means, respectively. We decided, a-priori, to conduct gender-stratified analyses due to expected differences in inequalities as reported in the literature [7, 8, 30]. Income-specific estimates were directly age-standardised within gender using the pooled data as standard. Pairwise differences between income groups (low-income households as reference) were evaluated on the absolute scale using a linear combination of the coefficients [31].

### Hurdle models

To handle continuous MVPA data with excess zeros and positive skewness, we used the hurdle model proposed by Cragg, which comprises two parts: a selection/participation model and a latent model [15]. The former determines the boundary points of the continuous outcome (a selection variable equals 1 if not bounded and 0 otherwise), whilst the latter determines its unbounded values (a continuous latent variable which is observed only if the selection variable equals 1). In our analyses, the selection model assessed the influence of income on the binary outcome of participation (any versus none), whilst the latent model assessed its influence on the amount of time spent active, conditional on participation (MVPA-active). We specified a probit model for the former and an exponential form for the latter. Each model contained income (as a three-category variable) and the confounders listed above.

Based on the model estimates, three sets of marginal means by income were calculated, evaluated at fixed values of the confounders. These sets correspond to different definitions of the expected value of MVPA [32]: (i) the probability of doing any, (ii) the average hours/week MVPA for all participants (the unconditional mean), including those who did none; and (iii) the average hours/week MVPA conditional on participation (MVPA-active). Inequalities after confounder adjustment (average marginal effects: AMEs) were quantified by computing the absolute difference in the marginal means (low-income as reference).

Dataset preparation and analysis was performed in SPSS V20.0 (SPSS IBM Inc., Chicago, Illinois, USA) and Stata V15.0 (College Station, Texas, USA), respectively. All analyses accounted for the complex survey design by applying sample weights (including correction for non-response) and incorporating the clustering of participants in postcode sectors (the primary sampling unit in the HSE series) via the “svy” package in Stata. HSE datasets are available via the UK Data Service (http://www.ukdataservice.ac.uk) [33–35]; statistical code is available from the corresponding author.

## Results

### Characteristics by income

Information on confounders by income is presented in Additional file 1. Poorer self-rated health and higher smoking levels were evident among adults in low-income households (both *P* < 0.001). BMI status also varied by income (P < 0.001 for both genders), with higher obesity levels especially among women in low-income households (Additional file 1).

### Descriptive estimates

**Fig. 1:**
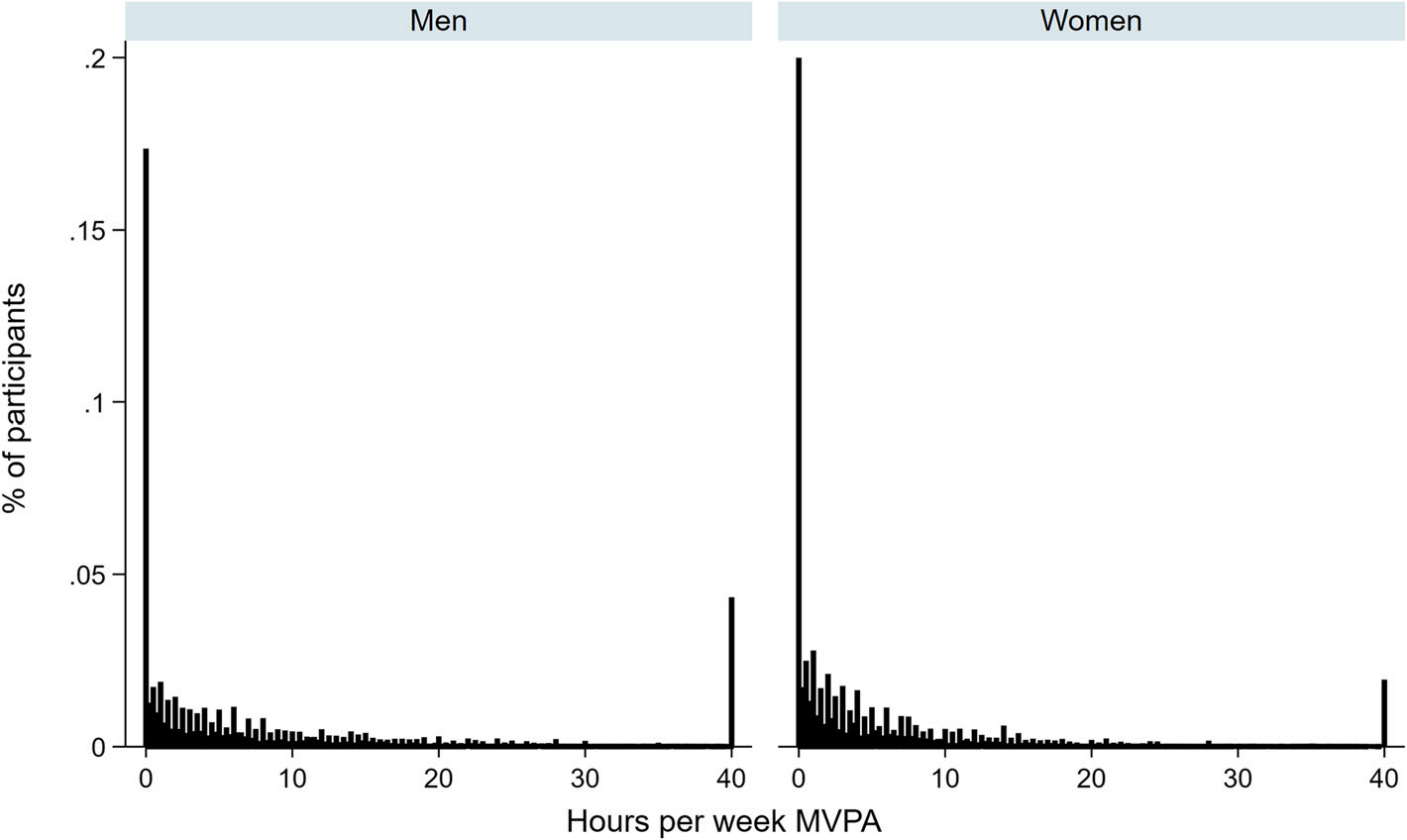
Distribution of hours per week spent in total MVPA by gender

Tables 1 and 2 show the descriptive estimates for total and domain-specific MVPA for all adults and by income for men and women, respectively. Overall, 85% of men (*n* = 9254) and 81% of women (*n* = 10,947) did any MVPA; 66% of men (*n* = 7120) and 56% of women (*n* = 7537) were ‘sufficiently’ active (Table 1 men; Table 2 women). Men and women spent on average 9.7 and 6.8 h/week respectively in total MVPA (Table 1 men; Table 2 women); however, these distributions showed excessive zeros and positive skewness (Fig. 1). Among those doing any MVPA, men and women spent on average 11.5 and 8.4 h/week respectively in total MVPA (Table 1 men; Table 2 women). The largest difference between MVPA and MVPA-active means was for occupational PA; among men, these were 2.5 and 15.2 h/week respectively (Table 1 men; Table 2 women).

**Table 1:** Total and domain-specific MVPA outcomes by income tertile among men, Health Survey for England 2008, 2012 and 2016

		Income						
	All	Lowest	Middle	Highest	Middle versus lowest		Highest versus lowest	
					Difference (95% CI)	***P***-value^**a**^	Difference (95% CI)	***P***-value^**a**^
N	11,199	3197	3729	4273				
**Total MVPA:**								
Any: % (95% CI)	85 (84, 85)	75 (73, 77)	86 (85, 87)	90 (89, 91)	11 (9, 13)	< 0.001	15 (13, 17)	< 0.001
Sufficient: % (95% CI)^b^	66 (65, 67)	54 (52, 56)	68 (66, 69)	74 (72, 75)	13 (11, 16)	< 0.001	19 (17, 22)	< 0.001
MVPA hours/week:mean (SE)^c^	9.7 (0.12)	8.1 (0.23)	10.3 (0.21)	10.4 (0.18)	2.2 (1.6, 2.8)	< 0.001	2.2 (1.7, 2.8)	< 0.001
MVPA-active hours/week:mean (SE)^d^	11.5 (0.13)	10.4 (0.27)	11.7 (0.23)	11.4 (0.19)	1.3 (0.6, 1.9)	< 0.001	0.9 (0.3, 1.6)	0.004
**Sports/exercise:**								
Any: % (95% CI)	53 (52, 54)	39 (37, 41)	52 (50, 53)	63 (61, 64)	13 (10, 15)	< 0.001	24 (21, 26)	< 0.001
Sufficient: % (95% CI)^b^	35 (34, 36)	24 (22, 25)	34 (32, 35)	42 (40, 43)	10 (8, 12)	< 0.001	18 (16, 20)	< 0.001
MVPA hours/week:mean (SE)^c^	3.5 (0.07)	2.4 (0.11)	3.3 (0.11)	4.3 (0.12)	0.9 (0.6, 1.2)	< 0.001	1.9 (1.6, 2.2)	< 0.001
MVPA-active hours/week:mean (SE)^d^	6.5 (0.10)	5.3 (0.20)	5.9 (0.16)	6.5 (0.16)	0.6 (0.0, 1.1)	0.032	1.2 (0.7, 1.7)	< 0.001
**Domestic:**								
Any: % (95% CI)	48 (47, 49)	43 (41, 45)	47 (45, 49)	52 (50, 54)	4 (1, 7)	0.003	9 (6, 11)	< 0.001
Sufficient: % (95% CI)^b^	10 (9, 10)	11 (10, 12)	9 (8, 10)	10 (9, 11)	-1 (−3, 0)	0.068	-1 (−3, 0)	0.086
MVPA hours/week:mean (SE)^c^	0.9 (0.03)	1.0 (0.06)	0.9 (0.04)	0.9 (0.04)	−0.1 (− 0.2, 0.1)	0.222	− 0.1 (− 0.3, 0.0)	0.070
MVPA-active hours/week:mean (SE)^d^	1.9 (0.05)	2.3 (0.13)	1.9 (0.08)	1.7 (0.06)	−0.4 (− 0.7, − 0.1)	0.006	−0.6 (− 0.9, − 0.3)	< 0.001
**Walking:**								
Any: % (95% CI)	43 (42, 44)	33 (31, 35)	40 (38, 42)	51 (49, 53)	7 (4, 10)	< 0.001	18 (16, 20)	< 0.001
Sufficient: % (95% CI)^b^	24 (24, 25)	20 (19, 22)	22 (20, 23)	30 (28, 31)	1 (−1, 3)	0.259	9 (7, 11)	< 0.001
MVPA hours/week:mean (SE)^c^	2.2 (0.05)	1.9 (0.10)	2.1 (0.09)	2.5 (0.09)	0.1 (−0.1, 0.4)	0.342	0.6 (0.3, 0.8)	< 0.001
MVPA-active hours/week:mean (SE)^d^	5.2 (0.11)	6.0 (0.27)	5.4 (0.22)	5.2 (0.19)	−0.5 (−1.2, 0.1)	0.109	−0.7 (− 1.4, − 0.1)	0.018
**Manual:**								
Any: % (95% CI)	28 (27, 29)	22 (20, 23)	29 (27, 30)	31 (29, 32)	7 (5, 9)	< 0.001	9 (7, 11)	< 0.001
Sufficient: % (95% CI)^b^	12 (11, 12)	9 (8, 10)	13 (12, 15)	12 (11, 13)	4 (3, 6)	< 0.001	3 (2, 5)	< 0.001
MVPA hours/week:mean (SE)^c^	1.1 (0.04)	0.8 (0.06)	1.3 (0.07)	1.1 (0.06)	0.4 (0.3, 0.6)	< 0.001	0.3 (0.2, 0.5)	< 0.001
MVPA-active hours/week:mean (SE)^d^	4.0 (0.10)	3.8 (0.23)	4.4 (0.22)	3.6 (0.17)	0.6 (−0.1, 1.2)	0.078	−0.2 (− 0.8, 0.3)	0.382
**Occupational:**								
In paid or unpaid work (%)^e^	64	35	65	83	–	–	–	–
Very physically active in their job^f^	25	38	31	16	–	–	–	–
Any % (95% CI)	17 (16, 17)	14 (13, 16)	22 (20, 23)	14 (13, 16)	7 (5, 9)	< 0.001	0 (−2, 2)	0.989
Sufficient % (95% CI)^b^	15 (14, 15)	13 (12, 15)	19 (18, 21)	12 (11, 13)	6 (4, 8)	< 0.001	−1 (−3, 1)	0.269
MVPA hours/week:mean (SE)^c^	2.5 (0.09)	2.4 (0.17)	3.6 (0.18)	1.9 (0.12)	1.1 (0.7, 1.6)	< 0.001	−0.5 (−0.9, − 0.1)	0.021
MVPA-active hours/week:mean (SE)^d^	15.2 (0.36)	16.3 (0.85)	15.7 (0.74)	13.6 (0.78)	−0.5 (−2.7, 1.7)	0.629	−2.7 (−4.9, − 0.4)	0.019

*MVPA* moderate-to-vigorous physical activity, *SE* standard error

^a^*P*-values calculated via linear combination of coefficients

^b^Sufficient activity: at least 2.5 h/week MVPA

^c^MVPA hours/week includes all participants, including those inactive (range: 0 to 40 h/week)

^d^MVPA-active hours/week restricted to active participants (range: 0.042 to 40 h/week)

^e^Estimates are unweighted

^f^Participants doing any paid or unpaid work were asked how physically active they were in their job (responses: very; fairly; not very; not at all). Estimates are unweighted

**Table 2:** Total and domain-specific MVPA by income tertile among women, Health Survey for England 2008, 2012 and 2016

	All	Lowest	Middle	Highest	Middle versus lowest		Highest versus lowest	
					Difference (95% CI)	***P***-value^a^	Difference (95% CI)	***P***-value^a^
N	13,683	4605	4627	4451				
**Total MVPA:**								
Any: % (95% CI)	81 (80, 82)	74 (73, 76)	81 (80, 82)	86 (85, 88)	7 (5, 8)	< 0.001	12 (10, 14)	< 0.001
Sufficient: % (95% CI)^b^	56 (55, 57)	49 (47, 50)	56 (54, 57)	63 (62, 65)	7 (5, 9)	< 0.001	14 (12, 16)	< 0.001
MVPA hours/week:mean (SE)^c^	6.8 (0.09)	5.8 (0.15)	6.9 (0.14)	7.6 (0.16)	1.1 (0.7, 1.5)	< 0.001	1.8 (1.3, 2.2)	< 0.001
MVPA-active hours/week:mean (SE)^d^	8.4 (0.10)	7.6 (0.17)	8.3 (0.16)	8.6 (0.17)	0.7 (0.3, 1.2)	0.001	1.0 (0.6, 1.5)	< 0.001
**Sports/exercise:**								
Any: % (95% CI)	44 (43, 45)	32 (30, 33)	43 (41, 44)	55 (54, 57)	11 (9, 13)	< 0.001	24 (22, 26)	< 0.001
Sufficient: % (95% CI)^b^	23 (22, 23)	15 (13, 16)	21 (20, 23)	30 (29, 32)	7 (5, 8)	< 0.001	16 (14, 18)	< 0.001
MVPA hours/week:mean (SE)^c^	2.0 (0.04)	1.3 (0.06)	1.8 (0.06)	2.8 (0.09)	0.5 (0.4, 0.7)	< 0.001	1.5 (1.3, 1.7)	< 0.001
MVPA-active hours/week:mean (SE)^d^	4.5 (0.08)	3.7 (0.13)	4.0 (0.12)	4.8 (0.15)	0.3 (0.0, 0.7)	0.051	1.1 (0.7, 1.5)	< 0.001
**Domestic:**								
Any: % (95% CI)	61 (60, 62)	60 (59, 62)	61 (60, 63)	60 (59, 62)	1 (−1, 3)	0.505	0 (−2, 2)	0.915
Sufficient: % (95% CI)^b^	20 (19, 20)	22 (21, 23)	20 (19, 22)	17 (16, 19)	−2 (−4, 0)	0.059	−5 (−6, −3)	< 0.001
MVPA hours/week:mean (SE)^c^	1.7 (0.03)	2.0 (0.07)	1.8 (0.06)	1.4 (0.05)	−0.2 (− 0.4, 0.0)	0.017	− 0.6 (− 0.7, − 0.4)	< 0.001
MVPA-active hours/week:mean (SE)^d^	2.8 (0.05)	3.2 (0.10)	2.8 (0.09)	2.3 (0.08)	− 0.3 (− 0.6, − 0.1)	0.013	− 0.8 (− 1.0, − 0.6)	< 0.001
**Walking:**								
Any: % (95% CI)	35 (34, 35)	27 (25, 28)	33 (32, 34)	43 (41, 44)	6 (4, 8)	< 0.001	16 (14, 18)	< 0.001
Sufficient: % (95% CI)^b^	22 (21, 23)	18 (16, 19)	21 (20, 23)	27 (25, 28)	4 (2, 5)	< 0.001	9 (7, 11)	< 0.001
MVPA hours/week:mean (SE)^c^	1.9 (0.05)	1.5 (0.07)	1.8 (0.07)	2.3 (0.09)	0.4 (0.2, 0.5)	< 0.001	0.8 (0.6, 1.1)	< 0.001
MVPA-active hours/week:mean (SE)^d^	5.5 (0.11)	5.6 (0.19)	5.8 (0.22)	5.8 (0.21)	0.2 (−0.4, 0.7)	0.526	0.2 (− 0.4, 0.7)	0.495
**Manual:**								
Any: % (95% CI)	12 (12, 13)	10 (9, 11)	12 (12, 13)	14 (13, 15)	2 (1, 3)	0.005	3 (2,5)	< 0.001
Sufficient: % (95% CI)^b^	4 (4, 4)	3 (3, 4)	4 (4, 5)	4 (4, 5)	1 (0, 2)	0.003	1 (0, 2)	0.009
MVPA hours/week:mean (SE)^c^	0.4 (0.02)	0.3 (0.02)	0.4 (0.03)	0.4 (0.03)	0.1 (0.0, 0.2)	0.008	0.1 (0.0, 0.2)	0.016
MVPA-active hours/week:mean (SE)^d^	2.9 (0.11)	2.7 (0.19)	3.1 (0.23)	2.5 (0.16)	0.4 (−0.2, 1.0)	0.177	−0.2 (− 0.7, 0.3)	0.408
**Occupational:**								
In paid or unpaid work (%)^e^	55	32	57	75	–	–	–	–
Very physically active in their job^f^	19	29	22	12	–	–	–	–
Any: % (95% CI)	7 (7, 7)	7 (6, 7)	9 (8, 9)	6 (6, 7)	2 (1, 3)	0.002	0 (−1, 1)	0.678
Sufficient: % (95% CI)^b^	6 (6, 6)	6 (5, 6)	8 (7, 8)	5 (5, 6)	2 (1, 3)	< 0.001	0 (− 1, 1)	0.658
MVPA hours/week:mean (SE)^c^	1.0 (0.04)	0.9 (0.08)	1.3 (0.08)	0.8 (0.07)	0.4 (0.1, 0.6)	0.001	−0.1 (− 0.3, 0.1)	0.329
MVPA-active hours/week:mean (SE)^d^	14.2 (0.39)	12.7 (0.66)	14.6 (0.65)	12.7 (0.59)	1.9 (0.1, 3.7)	0.043	−0.1 (−1.9, 1.7)	0.930

*MVPA* moderate-to-vigorous physical activity, *SE* standard error

^a^*P*-values calculated via linear combination of coefficients

^b^Sufficient activity: at least 2.5 h/week MVPA

^c^MVPA hours/week includes all participants, including those inactive (range: 0 to 40 h/week)

^d^MVPA-active hours/week restricted to active participants (range: 0.042 to 40 h/week)

^e^Estimates are unweighted

^f^Participants doing any paid or unpaid work were asked how physically active they were in their job (responses: very; fairly; not very; not at all). Estimates are unweighted

Inequalities were evident in descriptive analyses in each aspect for total MVPA and for sports/exercise. Differences between high-income versus low-income households in total MVPA were 2.2 h/week among men (95% CI: 1.7, 2.8; *P* < 0.001) and 1.8 h/week among women (95% CI: 1.3, 2.2; *P* < 0.001); the same pattern, but with narrower effect sizes, was found for total MVPA-active (men: 0.9 h/week, 95% CI: 0.3, 1.6; *P* = 0.004; women: 1.0 h/week, 95% CI: 0.6, 1.5; P < 0.001) (Table 1 men; Table 2 women). Likewise, differences in sports/exercise MVPA (i.e. including those who did none) for men and women in high-income versus low-income households were 1.9 h/week (95% CI: 1.6, 2.2; P < 0.001) and 1.5 h/week (95% CI: 1.3, 1.7; P < 0.001), respectively (Table 1 men; Table 2 women). Differences in sports/exercise MVPA-active were 1.2 h/week among men (95% CI: 0.7, 1.7; *P* < 0.001) and 1.1 h/week (95% CI: 0.7, 1.5; P < 0.001) among women (Table 1 men; Table 2 women).

Results for other domains were heterogeneous. Inequalities were evident in the unconditional outcomes (any; sufficient activity; MVPA) for walking, yet the time spent walking amongst those who did any walking was higher among men in low-income versus high-income households (levels were similar by income among women). Men in high-income versus low-income households did less occupational PA (MVPA: *P* = 0.021; MVPA-active: *P* = 0.019); whilst men (MVPA-active: *P* < 0.001) and women (P < 0.001 for MVPA and MVPA-active) in high-income households did less domestic activity (Table 1 men; Table 2 women).

### Multivariable hurdle models

**Fig. 2:**
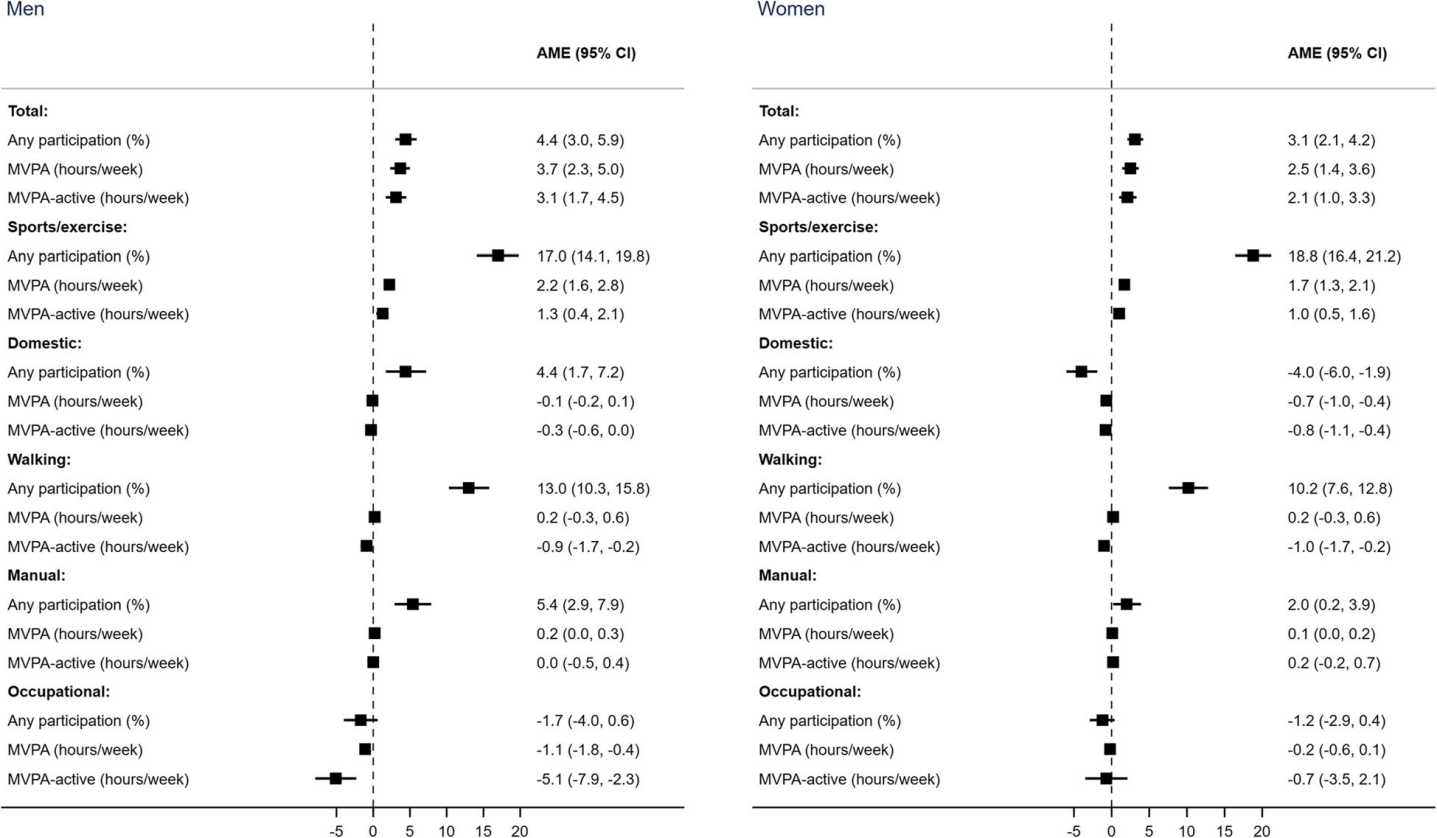
AMEs represent difference between adults in high-income versus low-income households in: (i) any participation (%); (ii) MVPA hours/week (average amongst all adults, including those who did none); and (iii) MVPA-active hours/week (average among those who did any). AMEs evaluated at fixed values of the confounders: for persons aged 35–44 years with very good/good health, never being a regular smoker, and having a normal-weight (BMI 18.5–24.9 kg/m^2^)

Table 3 shows the AMEs from estimated hurdle models corresponding to the absolute difference in the marginal means for the binary outome of participation, and the continuous outcomes of MVPA and MVPA-active (AMEs are graphically shown in Fig. 2).

**Table 3:** Parameter estimates from multivariable hurdle models (any participation and amount of time spent active), Health Survey for England 2008, 2012 and 2016

	Any (%)	***P***-value	Unconditional: Mean MVPA hours/week	***P***-value	Conditional: Mean MVPA-active hours/week	***P***-value
	AME (95% CI)^a^		AME (95% CI)^a^		AME (95% CI)^a^	
**Men**						
**Total:**						
Middle vs lowest	3.1 (1.9, 4.3)	< 0.001	2.7 (1.4, 4.0)	< 0.001	2.3 (0.9, 3.7)	0.001
Highest vs lowest	4.4 (3.0, 5.9)	< 0.001	3.7 (2.3, 5.0)	< 0.001	3.1 (1.7, 4.5)	< 0.001
**Sports/exercise:**						
Middle vs lowest	8.0 (5.1, 10.8)	0.002	0.9 (0.3, 1.5)	0.002	0.5 (−0.4, 1.4)	0.254
Highest vs lowest	17.0 (14.1, 19.8)	< 0.001	2.2 (1.6, 2.8)	< 0.001	1.3 (0.4, 2.1)	0.003
**Domestic:**						
Middle vs lowest	0.5 (−2.3, 3.3)	0.744	−0.1 (−0.2, 0.1)	0.260	−0.2 (− 0.5, 0.1)	0.187
Highest vs lowest	4.4 (1.7, 7.2)	0.002	−0.1 (− 0.2, 0.1)	0.314	− 0.3 (− 0.6, 0.0)	0.025
**Walking:**						
Middle vs lowest	3.2 (0.3, 6.1)	0.031	−0.3 (− 0.8, 0.1)	0.145	− 0.9 (− 1.7, − 0.2)	0.018
Highest vs lowest	13.0 (10.3, 15.8)	< 0.001	0.2 (− 0.3, 0.6)	0.430	− 0.9 (− 1.7, − 0.2)	0.015
**Manual:**						
Middle vs lowest	4.3 (1.8, 6.9)	0.001	0.3 (0.1, 0.5)	0.001	0.5 (0.0, 1.0)	0.059
Highest vs lowest	5.4 (2.9, 7.9)	< 0.001	0.2 (0.0, 0.3)	0.039	0.0 (−0.5, 0.4)	0.843
**Occupational:**						
Middle vs lowest	6.9 (4.5, 9.4)	< 0.001	1.3 (0.6, 2.0)	0.001	0.1 (−2.7, 2.9)	0.960
Highest vs lowest	−1.7 (−4.0, 0.6)	0.139	−1.1 (− 1.8, −0.4)	0.001	−5.1 (−7.9, − 2.3)	< 0.001
**Women**						
**Total:**						
Middle vs lowest	1.3 (0.4, 2.2)	0.004	1.1 (0.0, 2.2)	0.041	1.0 (−0.2, 2.2)	0.089
Highest vs lowest	3.1 (2.1, 4.2)	< 0.001	2.5 (1.4, 3.6)	< 0.001	2.1 (1.0, 3.3)	< 0.001
**Sports/exercise:**						
Middle vs lowest	8.3 (6.0, 10.7)	< 0.001	0.6 (0.2, 0.9)	0.001	0.2 (−0.3, 0.8)	0.431
Highest vs lowest	18.8 (16.4, 21.2)	< 0.001	1.7 (1.3, 2.1)	< 0.001	1.0 (0.5, 1.6)	< 0.001
**Domestic:**						
Middle vs lowest	−1.7 (−3.7, 0.2)	0.082	−0.3 (−0.6, − 0.1)	0.017	− 0.4 (− 0.7, 0.0)	0.045
Highest vs lowest	−4.0 (−6.0, − 1.9)	< 0.001	−0.7 (− 1.0, − 0.4)	< 0.001	−0.8 (− 1.1, − 0.4)	< 0.001
**Walking:**						
Middle vs lowest	2.9 (0.4, 5.3)	0.021	−0.1 (− 0.5, 0.3)	0.606	− 0.6 (− 1.3, 0.1)	0.107
Highest vs lowest	10.2 (7.6, 12.8)	< 0.001	0.2 (−0.3, 0.6)	0.487	− 1.0 (− 1.7, − 0.2)	0.012
**Manual:**						
Middle vs lowest	1.1 (−0.7, 3.0)	0.220	0.1 (0.0, 0.2)	0.093	0.3 (−0.2, 0.9)	0.231
Highest vs lowest	2.0 (0.2, 3.9)	0.032	0.1 (0.0, 0.2)	0.047	0.2 (−0.2, 0.7)	0.331
**Occupational:**						
Middle vs lowest	2.1 (0.5, 3.7)	0.010	0.6 (0.2, 1.0)	0.001	3.0 (0.3, 5.7)	0.030
Highest vs lowest	−1.2 (−2.9, 0.4)	0.132	−0.2 (−0.6, 0.1)	0.201	−0.7 (−3.5, 2.1)	0.620

*AME* average marginal effect

^a^Adjusting for age, self-rated health, smoking status and BMI status. Missing categories as additional category. AMEs evaluated at fixed values of the confounders: for persons aged 35–44 years with very good/good health, never being a regular smoker, and having a normal-weight (BMI 18.5–24.9 kg/m^2^)

Higher MVPA in high-income versus low-income households was robust to confounder adjustment for total MVPA and for sports/exercise (P < 0.001 for all outcomes and both genders; except *P* = 0.003 for sports/exercise MVPA-active among men) (Table 3). For example, at fixed values of the confounding variables, differences between high-income versus low-income households in sports/exercise MVPA were 2.2 h/week among men (95% CI: 1.6, 2.8) and 1.7 h/week among women (95% CI: 1.3, 2.1); differences in sports/exercise MVPA-active were 1.3 h/week (95% CI: 0.4, 2.1) and 1.0 h/week (95% CI: 0.5, 1.6) for men and women, respectively (Table 3).

Heterogeneity in associations was observed for other domains. Participants in high-income versus low-income households were more likely to do any walking (men: 13.0% (95% CI: 10.3, 15.8%); women: 10.2% (95% CI: 7.6, 12.8%)). Among all adults (including those who did no walking), the average hours/week spent walking showed no difference by income. Among those who did any walking, adults in high-income versus low-income households walked on average 1 h/week less (men: − 0.9 h/week (95% CI: − 1.7, − 0.2); women: − 1.0 h/week (95% CI: − 1.7, − 0.2)) (Table 3).

Women in high-income versus low-income households were less likely to do any (− 4.0%; 95% CI: − 6.0, − 1.9%, *P* < 0.001) and spent less time in domestic activity (P < 0.001 for MVPA and MVPA-active) (Table 3). Lower levels of occupational PA for men in high-income versus low-income households were robust to confounder adjustment (*P* = 0.001 and P < 0.001 for MVPA and MVPA-active) (Table 3).

## Discussion

Applying hurdle models to investigate inequalities in total and domain-specific MVPA, we hypothesised that adults in high-income households were more likely both to participate in MVPA than adults in low-income households and, conditional on doing any, to spend more time on average being active. These hypotheses were confirmed in fully-adjusted analyses for total MVPA and for sports/exercise. For example, among those doing any sports/exercise, men and women in high-income households spent on average 1.3 and 1.0 more hours/week in sports/exercise respectively, than their counterparts in low-income households (Table 3). Results for the other domains were mixed. Adults in high-income versus low-income households were more likely to do any walking. Among all adults (including those who did no walking), the average hours/week spent walking showed no difference by income. Among those who did any walking, adults in high-income versus low-income households walked on average 1 h/week less.

Comparisons with previous studies are difficult due to differences in study characteristics and analytical strategy. Bearing this caveat in mind, the inequalities in MVPA presented here agree with recent analyses of HSE data [7, 8], and with other European-wide [30, 36] and US [4, 6] studies. Our results showing that inequalities differ by domain corroborate both systematic reviews [5] and previous empirical studies [4], reflecting differences across SEP in how MVPA is accrued. In agreement with other reports [8], we found that inequalities in total MVPA were driven in the main by sports/exercise, which contributes a larger proportion of total MVPA for adults, especially men, in high-income households. This result also reflects inequalities in vigorous-intensity sports/exercise (data not shown), which is given twice the weight of moderate-intensity activities in our analyses in accordance with guidelines [26]. Inequalities in total and sports/exercise MVPA were partially offset by the reverse pattern for occupational PA, consistent with previous studies [4], reflecting the higher involvement of lower SEP groups in physically demanding work. Whilst occupational PA is taken into account in monitoring adherence to MVPA guidelines using HSE data [17, 18], high levels of strenuous occupational PA can be detrimental for health [37, 38].

Our findings add to the literature by assessing whether inequalities exist in the propensity to be active, in the amount of time spent active, or in both. Practitioners using the (unconditional) average to summarise inequalities should perform additional analyses to decompose this into its two parts: i.e. the probability of participation and the (conditional) average among those doing any [32]. Such decomposition can potentially shed light on the inequality determinants in the lower-tail of the distribution (drivers of inactivity) and those impacting the positive, non-zero, part of the distribution, implying potentially different tailored policy actions and interventions to reduce the gap in activity levels rather than a “one-size-fits-all” approach [7].

### Implications for policy

Differences in financial resources (especially for sports/exercise) [5] [39], health status [40], psychological or cultural characteristics [40, 41], and the built environment [40, 42, 43], including those driving inequalities in access to highly walkable neighbourhoods [44, 45], are key determinants of inequalities in physical activity. Reducing the inequalities presented here for sports/exercise will require policy actions and interventions to move adults in low-income households from inactivity to activity, and to enable those already active to do more. For example, removing user charges from leisure facilities in northwest England has had some success in increasing overall activity levels and in reducing inequalities [46]. Having world-class sports facilities that are free for anyone to use – as is the case in several Latin American cities – would reduce inequalities [47]. In contrast, our results suggest that interventions to promote walking should focus on reducing the sizeable income gap in the propensity to do any walking; such interventions could positively impact PA levels and reduce inequalities through increasing activity in the most sedentary [48] as well as the elderly and those in poorer health. A recent systematic review and meta-analysis [49] examined the effectiveness of interventions such as individual counselling [50], group training sessions [50, 51] and behavioural informatics [52] that were targeted at changing physical activity behaviour among low-income adults. The results showed a small positive intervention effect among those that focused on PA only as opposed to those targeting multiple behaviours [49]. However, evidence suggests that PA interventions are less effective in low-income groups, potentially widening rather than reducing inequalities [49]. Worryingly, a recent systematic review identified that there is insufficient evidence to allow for firm conclusions to be made regarding the impact of PA interventions on inequalities [53]. According to the WHO, effective national action to reduce disparities in PA requires a strategic combination of population-based policy actions aimed at tackling the “upstream” determinants that shape the equity of opportunities for participation (such as encouraging non-motorised modes of travel through improved provision of cycling and walking infrastructure, improved road safety, and creating more opportunities for PA in public open spaces and local community settings [54]) and those policy actions that are focused on “downstream” individually-focused (educational and informational) interventions, implemented in ways consistent with the principle of proportional universality (i.e. greatest efforts directed towards those least active) [55].

### Strengths and limitations

Our analyses used novel modelling methods to assess inequalities in MVPA. Although it is well-known that MVPA distributions typically contain excess zeros and positive skewness, no epidemiological studies to date have applied hurdle models to assess inequalities. Such models avoid the loss of information and power that occurs when practitioners typically categorise a continuous variable into a binary or ordinal variable [9]. Precision of our estimates was increased by pooling standardised PA data across survey years. Caution is required, however, when interpreting our findings. First, self-reported PA data has well-known limitations such as recall and reporting (social desirability) bias [56, 57]. Secondly, the dataset contained a sizeable amount of missing data for income and BMI (~ 20%); among HSE participants, the probability of having missing income data varies systematically across groups [58], which we minimised to some extent through applying non-response weights. The software routine for estimating hurdle models does not currently permit multiply imputed data, and so our findings may be statistically underpowered to some extent. Thirdly, the choice of potential confounders was limited to some extent by data availability; furthermore, we were unable to account for ethnic differences due to small numbers. As in all studies, our findings could have been influenced by unmeasured confounders. Fourthly, our findings are contingent upon HSE data collection, including the minimum duration of 10 min (in accord with the contemporaneous UK guidance but differing from recent UK [59] and US [60] guidelines, which acknowledge that PA of any duration enhances health), a specific subset of occupational PA for a selected group of occupations, and the inability to distinguish between walking for leisure and active travel. We acknowledge that different definitions may have led to different conclusions. Finally, we cannot draw causal inferences, as this was a descriptive study based on cross-sectional data.

### Future research

As mentioned previously, more evidence on the equitable impact of PA interventions is needed to ascertain ‘what works’ best to increase PA levels among low-income groups [49]. Given the aforementioned limitations of cross-sectional data on self-reported PA collected within large-scale national health examination surveys, it is imperative that innovative studies such as those using smartphones with built-in accelerometry to measure PA on a global scale [61] be used to shed light on inequalities and their interaction with aspects of the built environment such as walkability. However, maximising the potential for such research to inform policy-makers and practitioners will require efforts to minimise the potential bias of such data towards younger, more affluent, and more active populations [61]. Finally, as emphasised in this study, whatever the source of data, separate model equations should be used to assess inequalities in participation and in duration.

## Conclusion

Monitoring inequalities in MVPA requires assessing different aspects of the distribution within each domain. In the present study, income-based inequalities were evident in the propensity to do any sports/exercise and walking, and for the amount of time spent doing sports/exercise. These findings may assist policy-makers to identify and commission tailored interventions best suited to tackling inequalities, and our methods could be used by practitioners to evaluate their impact.

## Supplementary information

**Additional file 1:** Distribution of participants on the key variables by income tertile and gender, Health Survey for England 2008, 2012 and 2016.

## Data Availability

The HSE datasets generated and/or analysed during the current study are available via the UK Data Service (UKDS: https://ukdataservice.ac.uk/). Syntax to enable replication of our results (using the datasets deposited at the UKDS) is available on request from the corresponding author.

## Abbreviations

AME: Average marginal effects
BMI: Body mass index
HSE: Health Survey for England
MVPA: Moderate-to-vigorous physical activity
PA: Physical activity
PASBAQ: Physical activity and sedentary behaviour assessment questionnaire
SEP: Socio-economic position
WHO: World Health Organization
