# Supplementary material for: Inequalities in participation and time spent in moderate-to-vigorous physical activity: a pooled analysis of the cross-sectional health surveys for England 2008, 2012, and 2016
Source: BMC Public Health. 2020 Mar 19;20:361. doi: 10.1186/s12889-020-08479-x (PMC7082987; doi:10.1186/s12889-020-08479-x)
Supplement: Supplementary file 1 — Additional file 1: Distribution of participants on the key variables by income tertile and gender, Health Survey for England 2008, 2012 and 2016. [file 12889_2020_8479_MOESM1_ESM.docx]

**Additional File 1: Key variables by income tertile and gender, Health Survey for England 2008, 2012 and 2016**

|  | **Men** | | | | **P-value^a^** |  | **Women** | | | | **P-value^a^** |
| --- | --- | --- | --- | --- | --- | --- | --- | --- | --- | --- | --- |
|  |  | **Income** | | |  |  |  | **Income** | | |  |
|  | **All** | **Lowest** | **Middle** | **Highest** |  |  | **All** | **Lowest** | **Middle** | **Highest** |  |
|  | **N (%)** | **N (%)** | **N (%)** | **N (%)** |  |  | **N (%)** | **N (%)** | **N (%)** | **N (%)** |  |
| **All** | 11199 (100) | 3197 (100) | 3729 (100) | 4273 (100) | - |  | 13683 (100) | 4605 (100) | 4627 (100) | 4451 (100) | - |
| **Age-group:** |  |  |  |  |  |  |  |  |  |  |  |
| 16-34 | 2695 (31) | 799 (32) | 865 (30) | 1031 (30) | <0.001 |  | 3556 (30) | 1276 (32) | 1131 (29) | 1149 (30) | <0.001 |
| 35-54 | 3909 (37) | 829 (29) | 1225 (36) | 1855 (44) |  |  | 4942 (36) | 1345 (30) | 1620 (35) | 1977 (43) |  |
| 55-74 | 3527 (25) | 1051 (26) | 1272 (27) | 1204 (23) |  |  | 3864 (25) | 1294 (25) | 1418 (27) | 1152 (23) |  |
| 75+ | 1068 (7) | 518 (12) | 367 (7) | 183 (3) |  |  | 1321 (9) | 690 (14) | 458 (9) | 173 (3) |  |
| **Smoking:** |  |  |  |  |  |  |  |  |  |  |  |
| Never | 5349 (50) | 1199 (40) | 1765 (50) | 2385 (57) | <0.001 |  | 7817 (58) | 2289 (51) | 2602 (57) | 2926 (66) | <0.001 |
| Ex-regular | 3443 (27) | 1023 (28) | 1227 (29) | 1193 (26) |  |  | 3208 (22) | 1017 (21) | 1166 (24) | 1025 (22) |  |
| Current | 2327 (22) | 938 (31) | 717 (20) | 672 (17) |  |  | 2573 (19) | 1258 (27) | 832 (18) | 483 (11) |  |
| **Self-rated health:** |  |  |  |  |  |  |  |  |  |  |  |
| Very good/good | 8379 (77) | 1890 (63) | 2808 (78) | 3681 (87) | <0.001 |  | 10185 (76) | 2854 (63) | 3478 (76) | 3853 (87) | <0.001 |
| Fair | 2033 (17) | 815 (24) | 714 (18) | 504 (11) |  |  | 2483 (17) | 1173 (24) | 834 (18) | 476 (10) |  |
| Bad/very bad | 785 (6) | 490 (14) | 207 (5) | 88 (2) |  |  | 1012 (7) | 576 (12) | 314 (6) | 122 (3) |  |
| **BMI status:** |  |  |  |  |  |  |  |  |  |  |  |
| Underweight | 117 (1) | 61 (2) | 33 (1) | 23 (1) | <0.001 |  | 209 (2) | 85 (2) | 67 (2) | 57 (1) | <0.001 |
| Normal | 2858 (28) | 820 (28) | 936 (28) | 1102 (27) |  |  | 4581 (35) | 1332 (30) | 1498 (33) | 1751 (40) |  |
| Overweight | 4295 (37) | 1102 (33) | 1420 (37) | 1773 (41) |  |  | 3733 (27) | 1179 (25) | 1342 (28) | 1212 (27) |  |
| Obese | 2511 (21) | 719 (21) | 866 (22) | 926 (21) |  |  | 3104 (22) | 1271 (27) | 1036 (22) | 797 (18) |  |
| Missing | 1418 (12) | 495 (15) | 474 (12) | 449 (11) |  |  | 2056 (15) | 738 (16) | 684 (15) | 634 (14) |  |

BMI: Body mass index. Figures are column percentages: missing data for smoking and self-rated health not shown. Sample sizes are unweighted; percentages are weighted but are not age-standardised. ^a^ P-values obtained using Rao-Scott tests for independence in 2-way tables; P values obtained excluding missing category.
